# Novel Primer Sets for Next Generation Sequencing-Based Analyses of Water Quality

**DOI:** 10.1371/journal.pone.0170008

**Published:** 2017-01-24

**Authors:** Elvina Lee, Maninder S. Khurana, Andrew S. Whiteley, Paul T. Monis, Andrew Bath, Cameron Gordon, Una M. Ryan, Andrea Paparini

**Affiliations:** 1 Vector- and Water-Borne Pathogen Research Laboratory, School of Veterinary and Life Sciences, Murdoch University, Perth, Western Australia, Australia; 2 School of Earth and Environment, The University of Western Australia, Crawley, Western Australia, Australia; 3 Australian Water Quality Centre, South Australian Water Corporation, Adelaide, South Australia, Australia; 4 Drinking Water Quality Branch, Water Corporation, Leederville, Western Australia, Australia; Agricultural University of Athens, GREECE

## Abstract

Next generation sequencing (NGS) has rapidly become an invaluable tool for the detection, identification and relative quantification of environmental microorganisms. Here, we demonstrate two new 16S rDNA primer sets, which are compatible with NGS approaches and are primarily for use in water quality studies. Compared to 16S rRNA gene based universal primers, *in silico* and experimental analyses demonstrated that the new primers showed increased specificity for the Cyanobacteria and Proteobacteria phyla, allowing increased sensitivity for the detection, identification and relative quantification of toxic bloom-forming microalgae, microbial water quality bioindicators and common pathogens. Significantly, Cyanobacterial and Proteobacterial sequences accounted for ca. 95% of all sequences obtained within NGS runs (when compared to ca. 50% with standard universal NGS primers), providing higher sensitivity and greater phylogenetic resolution of key water quality microbial groups. The increased selectivity of the new primers allow the parallel sequencing of more samples through reduced sequence retrieval levels required to detect target groups, potentially reducing NGS costs by 50% but still guaranteeing optimal coverage and species discrimination.

## Introduction

The growing accessibility of next generation DNA sequencing (NGS) methods has greatly advanced our understanding of microbial diversity in medical and environmental science [1–5]. Refinement of platforms, protocols and reagents for parallel high-throughput DNA sequencing technologies allows profiling of complex microbial communities at ever-increasing resolution [6, 7]. Being culture independent, NGS has the advantage of allowing direct analysis of communities as they exist under *in situ* conditions, including their genes, transcripts, proteins, and metabolites and how their reciprocal interactions impact their distribution patterns [6, 8, 9]. Microbiomes can be audited with or without the preliminary use of polymerase chain reaction (PCR) amplification of DNA; the sequences, from either selected loci, or entire genomes [10, 11] are then taxonomically or functionally classified based on the similarity to known entries from existing databases [7].

The success and resultant quality of PCR-based sequencing can be affected by factors such as the amplicon targeted (locus and region), thermal cycling conditions used, sequencing method, bioinformatics pipeline etc. [6, 7, 12–14]; yet, the most critical step in amplification-dependent metagenomics studies still appears to be amplification region targeted and the choice of primers [15–18]. Compared to other loci, the hypervariability and multi-copy nature of the small ribosomal subunit RNA (16S rDNA) gene, coupled with the availability of growing sequence information, confer higher detectability and allow taxonomic classification of bacteria and archaea, potentially to species level [19].

Previous research has concentrated on the design of 16S rDNA primers that amplify all taxa with equal efficiency [16, 20, 21] to guarantee broad taxonomic representation and preserve the relative proportions originally present in the community [17, 20, 22–24]. 16S rDNA-targeted primers with broad taxonomic coverage (known as “universal” primers) are routinely employed for exhaustive molecular surveys of bacterial and archaeal communities. At inadequate sequencing depths and in the presence of strong dominance, however, universal primers are expected to generate a large proportion of the sequences from the most abundant taxa, while rarer taxa (possibly including pathogens, bioindicators or other informative target groups) may remain undetected [25].

In the context of the water quality, the utilisation of NGS has allowed for the discovery of new indicators of water contamination (as reviewed in [26]). Similarly, cyanobacteria are a global concern for the water industry, due to the presence of bloom forming and/or secondary metabolite producing strains that are a risk to public health or adversely affect the aesthetic quality of the water [27–29]. Molecular detection, quantification and identification of cyanobacteria are relatively common [8, 30–33], but the numerous primer sets currently available generate amplicons with lengths incompatible with NGS, and/or are specific for only a few members of this phylum [34–40]. To overcome these limitations, the present study sought to design taxon-specific primers targeting the cyanobacteria 16S rRNA gene for use with NGS. These new primers were tested on both cultures and environmental water samples; the results were compared against a highly validated universal primer set, to determine the potential utility of the novel primers from the current study, in the context of water quality monitoring.

## Materials and Methods

### Cyanobacteria cultures, sampling and DNA extraction

Two types of water sources were used in this study: Cyanobacterial cultures isolated in a previous study [33], and heterogeneous environmental samples obtained from a variety of public sites and unrestricted ecosystems around Perth, WA (Table 1). These water samples were obtained with the knowledge of the relevant municipal authorities. Field campaigns were conducted in open spaces accessible to the public and no permissions were required. Field campaigns did not involve any interaction with endangered or protected species or ecosystems. Urban freshwater samples (1 L) were collected in the winter of 2014 (Piney Lakes Reserve) or summer 2015. Grab samples (1 L) were collected in the winters of 2014 and 2015, from the final effluents of three wastewater treatment plants (WWTP) in Western Australia. These WWTP collection sites had previously been selected and sampled with the assistance and permission from the managing authority (WaterCorporation of WA). to cover different construction design, treatment technologies, and operating regimes. Extractions of DNA from Cyanobacterial cultures (n = 6) were performed as previously described [41]. Whole DNA was extracted from environmental water samples using the MOBIO PowerWater Sterivex DNA Isolation kit (MOBIO, USA) according to the manufacturer’s instructions (n = 10). Extraction blanks (n = 2) were always included in every batch of DNA extractions; these consisted of mock-extractions from only fresh reagents (for extractions from cell cultures) or a fresh Sterivex filter (for extractions from water samples). All reactions were carried out using DNA-free reagents and consumables, with amplification and sequencing reactions set up in a dedicated laboratory area, physically isolated from post-amplification areas and free of PCR products.

**Table 1:** Environmental water samples collection sites.

Site	Approx. GPS Location	Sample type
Blue Gum Lake	-32.0408, 115.8467	Urban freshwater
Booragoon Lake	-32.0443, 115.8415	Urban freshwater
Frederick Baldwin Park Lake	-32.0594, 115.8131	Urban freshwater
Marmion Reserve Lake	-32.0390, 115.8114	Urban freshwater
Murdoch University Chinese Garden Koi Pond	-32.0667, 115.8324	Urban freshwater
Piney Lakes Reserve Lake	-32.0497, 115.8381	Urban freshwater
Alfred Cove Drain	-32.0291, 115.8126	Urban runoff
WWTP1	North West region of WA*	Wastewater treatment plant final effluent
WWTP2	South-West region of WA*	Wastewater treatment plant final effluent
WWTP3	South-West region of WA*	Wastewater treatment plant final effluent

*exact location is confidential.

### Primer design and *in silico* validation

Cyanobacteria-specific primers separately targeting the V3 and V6 regions (Locations indicated in S1 Fig) of the 16S rDNA were designed using the Primer 3 [42] add-on in Geneious Pro 8.0.4 [43]. The primers were designed from an alignment of 6,513 cyanobacteria 16S rDNA sequences retrieved from NCBI GenBank (April 2014), with lengths ranging from 144 to 1,481 bp. The alignment was constructed in Geneious Pro 8.0.4 [43], using the MAFFT plugin [44] after discarding non-overlapping sequences. Primers were designed using target consensus sequences generated for a range of stringency levels (e.g., 40% -75% consensus). The sequences and regions targeted by the primers used in this study are shown in Table 2. *In silico* tests of primer specificity were conducted using NCBI BLAST, TestPrime 1.0 (http://www.arb-silva.de/search/testprime/) and ProbeMatch (https://rdp.cme.msu.edu/probematch) and the SILVA and Ribosomal Database Project (RDP) databases. The settings chosen for the SILVA analysis by TestPrime 1.0 were: Maximum number of mismatches: 1; length of 0-mismatch zone at 3' end: 5 bases; SILVA Database: SSURef-122 NR. One and two differences were allowed for the RDP analysis by ProbeMatch.

**Table 2:** List of primers used in the present study, region targeted and amplification properties.

Primer name	Sequence (5’-3’)	Location on *E*.*coli* K-12 (NR102804)	Target region	Annealing temperature	Approximate amplicon size	Reference
293F	AGCCACACTGGGRCTGAGA	312–331	V3	50°C	255 bp	This study
751R	TGCGGACGCTTTACGCCCA	572–590				
515F	GTGCCAGCMGCCGCGGTAA	523–541	V4	55°C	253 bp	[20]
806R	GGACTACHVGGGTWTCTAAT	796–816				
1328F	GCTAACGCGTTAAGTATCCCGCCTGG	870–896	V6	55°C	298 bp	This study
1664R	GTCTCTCTAGAGTGCCCAACTTAATG	1166–1185				

### Library preparations and NGS

During the present study, the designed primers were used together with a universal primer pair, targeting the V4 hypervariable region of the 16S rRNA gene, amplified with the modified versions [45] of the 515F_806R primers [20], following the protocol version 6_15 (http://www.earthmicrobiome.org). For the generated primers, thermal cycling conditions were optimized in 25 μL reactions containing 1U of PerfectTaq DNA polymerase (5 Prime, Germany), 2 mM MgCl_2_, 200 μM of each deoxynucleotide triphosphate (dNTP, Promega), 0.2 μM of each primer and 1 μL (approx. 30ng) DNA template obtained either during a previous study [33]. Gradient PCR protocols were implemented in a Veriti 96-well Thermal Cycler (Applied Biosystems) with annealing temperatures ranging from 50°C to 60°C. Amplicons were visualised on a 2% agarose gel.

With fusion primers (IDT, USA), three separate amplification reactions were carried out per primer, for each sample; reaction mixtures were the same as above, but also contained 0.01 mg BSA (Fisher Biotech, Australia) and 3.3 μM SYTO 9 (Thermo Fisher Scientific, USA). No-template-controls (NTC) and extraction blanks were included in every experiment. Thermal cycling involved an initial denaturation at 95°C for 5 mins, followed by 45 cycles of denaturation at 95°C for 30 s, annealing at the appropriate temperature for 30 s, and extension at 72°C for 45 s and a final extension at 72°C for 7 min, which was followed by a melting curve analysis in a Step-One real-time qPCR instrument (Applied Biosystems, USA). Chosen annealing temperatures were 50°C and 55°C for 293F_751R and 1328F_1664R, respectively.

Multiplexing was based on six-to-eight bp multiplex identifier (MID) sequences (Thermo Fisher Scientific, USA); a unique combination of forward and reverse MID sequences was used for each sample.

To obtain the final library, amplicons from all samples and controls were pooled in equimolar amounts, based on the Ct values obtained from each sample by qPCR-based relative quantifications. This was purified twice using 1.2 volumes of Agencourt Ampure XP beads (Agilent Technologies, USA) and quantified by qPCR using a known concentration of a serially diluted 152 bp synthetic oligonucleotide as a standard. For qPCR-based absolute quantifications, reactions contained approximately 50 pg of library, Power SYBR Green Master Mix (Thermo Fisher Scientific, USA), 0.4 μM of each primer targeting the A and P1 Ion Torrent adapters (IDT, USA); an annealing temperature of 60°C was chosen.

Emulsion PCR and enrichment were performed according to the manufacturer’s recommendations on the One-Touch 2 and One-Touch ES instruments (Thermo Fisher Scientific, USA). Sequencing was performed on an Ion Torrent PGM (Thermo Fisher Scientific, USA) using 400 bp chemistry and 316-V2 semiconductor chips, following the manufacturer’s recommendations.

### Bioinformatics analysis

Sequences were first processed in Geneious Pro 8.0.4 [43] by retaining only reads with perfect matches with the forward- and reverse-primers and MID sequences (no mismatches allowed). Sequences were then de-multiplexed into individual samples based on their unique combination of MID sequences. Primers were trimmed from each read, and sequences were filtered based on length by discarding all reads shorter than 200 bp. All sequences obtained with the same primer set, from all samples, were renamed and grouped, prior to further analysis. At this stage, there were 381,504, 950,040 and 284,297 sequences for 293F_751R, 515F_806R and 1328F_1664R, respectively. The reads were then quality-filtered using USEARCH [46], allowing only reads with a < 1% error rate to remain and singletons were removed on a per-sample basis. In order to have equal sampling depth for each primer set, every sample was then subsampled to an equal number of sequences in QIIME [47].

Operational taxonomic units (OTUs) were selected by clustering sequences at 97% similarity with the UPARSE algorithm [48], and bacterial genera present were identified. OTUs were checked against the ChimeraSlayer Gold reference database with the UCHIME algorithm [49] to ensure OTUs were not the result of chimeric reads. Taxonomy was assigned to OTUs against the GreenGenes 16S rRNA gene database (August 2013 release) [50] in QIIME 1.9.0 [47] using the UCLUST algorithm [46] with default parameters. Sequences identified in extraction blanks and NTCs were bioinformatically removed from each sample in which they appeared, to eliminate background contamination. Diversity analyses were then performed, with the number of sequences analysed for each primer set determined by the sample with the least number of sequences (for rarefaction).

16S rDNA sequences from the most abundant phyla obtained by all three primer sets (i.e., from Cyanobacteria and Proteobacteria), were compared against the NCBI GenBank nucleotide database, using the BLAST algorithm to obtain increased taxonomic resolution. The results were then visualised using the MEtaGenome ANalyzer (MEGAN) version 5 [51] (default settings except *Min Score* = 150 and *Min Complexity* = 0.3).

## Results

### *In silico* PCR

The results from both the TestPrime and ProbeMatch *in silico* analyses indicated that, compared to the universal primers pair (515F_806R) [20], the novel primers 293F_751R and 1328F_1664R targeted at least three times more Cyanobacterial sequences (Table 3). The TestPrime analyses showed that Cyanobacterial sequences formed 15.0% and 95.0% of the total sequences amplified by the 293F_751R and 1328F_1664R primer pairs respectively, as compared to the universal 515F_806R for which Cyanobacterial sequences comprised only 2.0%. This increased specificity for Cyanobacterial sequences was also seen in the analyses performed using ProbeMatch (Table 3).

**Table 3:** *In silico* analysis showing the fraction (%) of Cyanobacterial sequences, from the sequences present in the SILVA and RDP databases, that are amplified by the primer pairs 293F_751R, 515F_806R and 1328F_664R.

Primer pair	SILVA	RDP	
		1 difference allowed	2 differences allowed
293F_751R	15.0%	23.0%	12.0%
515F_806R	2.0%	4.0%	4.0%
1328F_664R	95.0%	95.0%	60.0%

**Fig 1:**
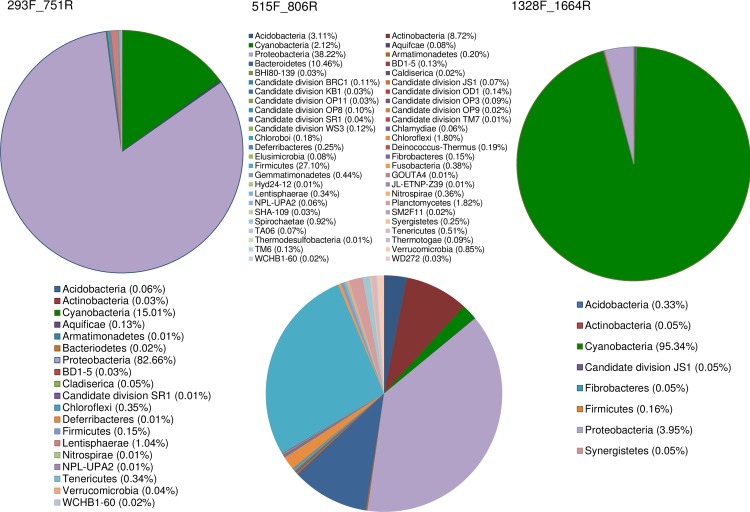
Pie charts showing the relative proportion of phyla amplified by each primer set used in the present study, as predicted by *in silico* PCR tests. *In silico* tests were performed against the SILVA database. For clarity, phyla forming less than 0.01% of the predicted amplifications were omitted.

Based on *in silico* PCR analyses and compared to primers 515F_806R [20], the novel primers 293F_751R and 1328F_1664R showed higher taxonomic specificity by targeting a narrower range of phyla available in the SILVA database (Fig 1). The 293F_751R and 1328F_1664R primer pairs, provided matches for 19 and 7 main phyla respectively, compared to 50 phyla for 515F_806R.

The phylum Cyanobacteria included the largest fractions of the predicted target sequences of the two novel primer sets, compared to the universal primers: 15.0% (293F_751R) and 95.3% (1328F_1664R) compared to 2.1% (515F_806R). Primers 293F_751R appeared also highly specific for Proteobacteria, as compared to 515F_806R (82.7% vs. 38.2% of the predicted target sequences).

### Community analysis by NGS

**Fig 2:**
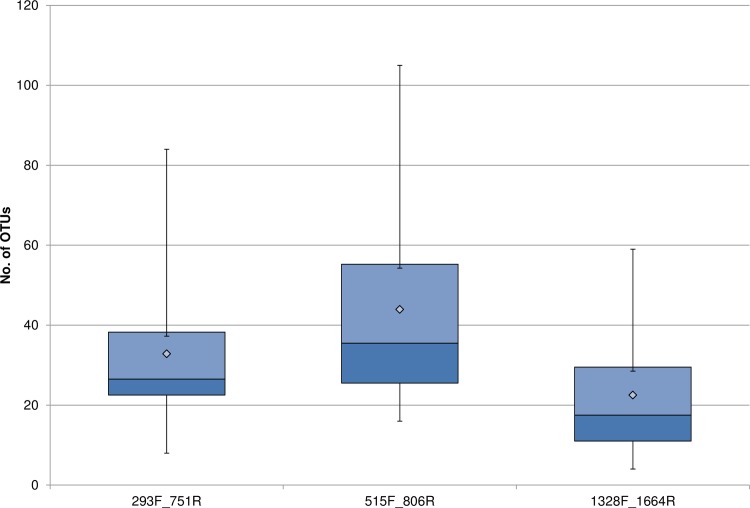
Box plot showing the alpha diversity values observed from all samples amplified by each primer set. Grey diamonds represent the average number of OTUs observed by each primer pair; boxes give minimum, first quartile, median, third quartile, and maximum, for each primer pair.

**Fig 3:**
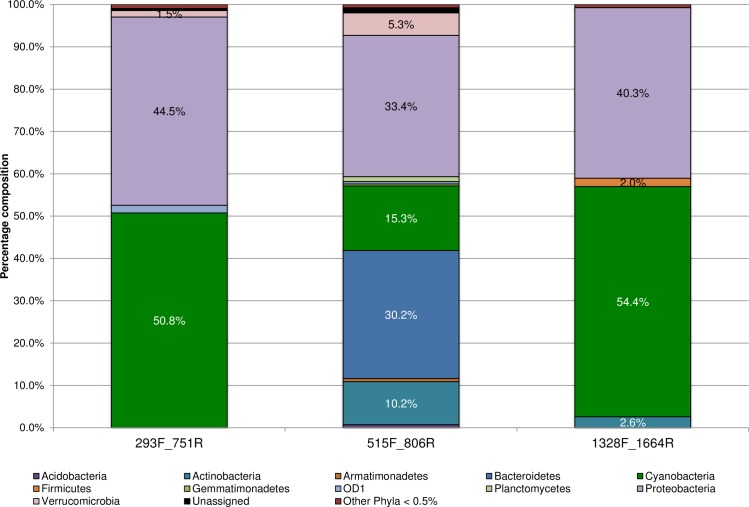
Percentage composition of sequences belonging to the indicated phyla, amplified by each primer set used in the present study. Subsampling was carried out to allow equal sequencing depth across all samples (~27,325 reads/sample) and to facilitate comparisons. For clarity, phyla forming < 0.5% abundance for each primer pair were combined.

After *in silico* PCR, the novel primers were then tested by NGS on several environmental samples (Table 1) to confirm their utility and to determine optimal amplification conditions. The sequence and final optimal amplification conditions used for the primers designed in this study are given in Table 2. After quality filtering, a total of 339,008, 852.293 and 237,860 sequences remained for the 293F_751R, 515F_806R, and 1328F_1664R primer pairs respectively (BioProject ID: PRJNA330773). Subsampling resulted in the selection of 27,325 sequences per sample, from each of the three primer sets (Rarefaction analysis showing sampling depth shown in S2 Fig). Sample sequences were pooled by primer pair, which allowed for data comparisons across primers. In agreement with the *in silico* analyses, after exclusion of background contaminations and OTUs, which were either unassigned or classified only to the bacterial root, samples amplified with the universal 515F_806R primers had the greatest diversity of genetic variants within each sample (alpha diversity), while those with 1328F_1664R had the smallest (average of 44 OTUs vs. 23 OTUs respectively) (Fig 2). The increased specificity of the novel primers is also reflected in the number of bacterial phyla obtained by NGS (Fig 3). The two novel primer sets seem to perform similarly to each other, especially compared to what was predicted *in silico*. The 293F_751R, 515F_806R, and 1328F_1664R primers detected a total of 8, 14 and 7 bacterial phyla respectively and also successfully amplified members of the phylum Proteobacteria. Altogether, Proteobacteria and Cyanobacteria formed approximately 95.0% of the subsampled sequences obtained by both the 293F_751R and 1328F_1664R primer pairs, as opposed to the universal 16S rDNA primers, where these two groups comprised less than half (48.7%) of the sequences (Fig 3). Other common environmental phyla were also detected and formed various proportions of the subsampled data for each primer set (e.g., Actinobacteria, Verrucomicrobia, Firmicutes, Bacteroidetes etc.). These bacterial groups, however, were only represented as minor fractions (≤ 2.6%) of the taxa audited by the 293F_751R and 1328F_1664R primer pairs. In contrast, the broader specificity of the universal 16S rDNA primers 515F_806R [20] allowed greater amplification of sequences from these phyla, which formed at least 5.3% relative abundance (Fig 3, detailed results in S1 File).

**Fig 4:**
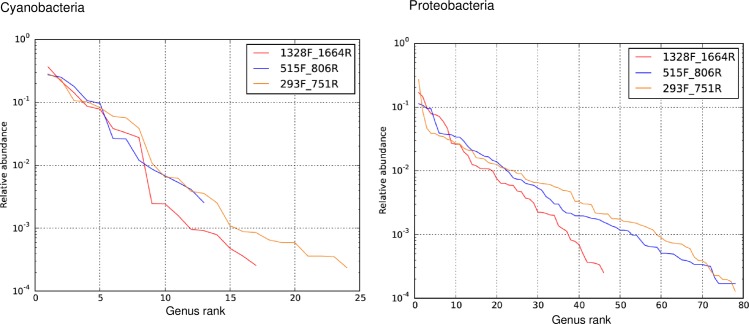
Comparison of taxonomic coverage of Cyanobacteria and Proteobacteria by primer sets.

The potential of the primers to taxonomically identify sequences to a lower (genus) level was compared using rank abundance curves (Fig 4). For the phylum Cyanobacteria, the primer set 293F_751R had the largest number of successfully assigned organisms, followed by 1328F_1664R and the universal 16S rDNA primers 515F_806R. For the identification of members of the Proteobacteria phylum, the 293F_751R and the universal 16S rDNA primers performed similarly (Fig 4).

Comparison of the results for each phyla showed that each of the primer sets appeared to preferentially amplify different orders from within the Cyanobacteria and Proteobacteria phyla. For Cyanobacteria, the 293F_751R primer set amplified all (9/9) of the 4C0d-2/Chloroplast (class), all (5/5) of the Pseudanabaenales (order), and 3/5 of the Oscillatoriophycideae (class) (1 Oscillatoriales and 2 Chroococcales (class)) observed. For 1328F_1664R, only Cryptophyta (order) was amplified from the 4C0d-2/Chloroplast class; 4/6 of the Nostocales (order), 4/5 of the Pseudanabaenales (order), all the Chroococcales (order) (3/3) and all of the Synechococcales (order) (2/2) observed were amplified. In comparison, the universal 16S rDNA primers amplified the smallest range of Cyanobacteria (cf. Fig 4, S2 File), amplifying 5/9 of 4C0d-2/chloroplast (class), 3/6 of the Nostocales and 2/5 of the Pseudanabaenales class/orders, a single genus from the Oscillatoriophycideae (class) was amplified, while no amplification was obtained for the Synechoccales (order) and Chroococcales (order). For the Proteobacteria, a similar pattern was also observed with the different primer sets having different degrees of amplification success for the various Proteobacterial orders (as illustrated in S3 File).

BLAST-searches were performed to further test the ability of the primers for detecting taxa of potential health and environmental concern. Human, animal and plant pathogens, as well as toxic bloom-forming Cyanobacterial species were detected by all primers in the water samples collected during the present experiments (Table 4). For instance, the 293F_751R primer pair demonstrated the potential to identify the presence of pathogenic species or genera such as *Neisseria canis*, *Coxiella burnetii*, the causative agent for Q fever [52], and *Ralstonia* sp., *Burkholderia* sp. (genera including plant and human pathogens). This demonstrates the potential usefulness of the novel primers in future DNA-based applications towards water quality monitoring.

**Table 4:** Illustrative comparison of taxa of potential health and environmental concern as detected by the various primer pairs, in the water samples collected during the present study. Classifications are given according to the lowest-level taxonomic assignments obtained, by bioinformatics analyses. Listed genera include those containing medically important organisms of humans and animals and toxic bloom-forming microalgae.

Phylum	Proteobacteria			Cyanobacteria		
Primer Set	293F_751R	515F_806R	1328F_1664R	293F_751R	515F_806R	1328F_1664R
	*Alcaligenes* sp.	*Acrobacter* sp		*Calothrix* sp.	*Calothrix* sp.	*Anabaena oscillariodes*
	*Aeromonas* sp.	*Coxiella burnetii*		*Limnothrix planktonica*	*Planktothrix* sp.	*Aphanizomenon gracile*
	*Burkholderia* sp.	*Pasteurella testundinis*		*Phormidium* sp.	*Trichormus variabilis*	*Limnothrix* sp.
	*Coxiella burnetii*	*Pseudomonas* sp.		*Planktothrix* sp.		*Nostoc linckia*
	*Endozoicomonas elysicola*	*Rickettsiales* sp.		*Trichormus variabilis*		*Phormidium* sp.
	*Nesseria canis*					*Pseudanabaena gelata*
	*Ralstonia* sp.					*Pseudanabaena limnetica*
						*Sphaerospermopsis aphanizomenoides*

### Microbial community in environmental water samples

**Fig 5:**
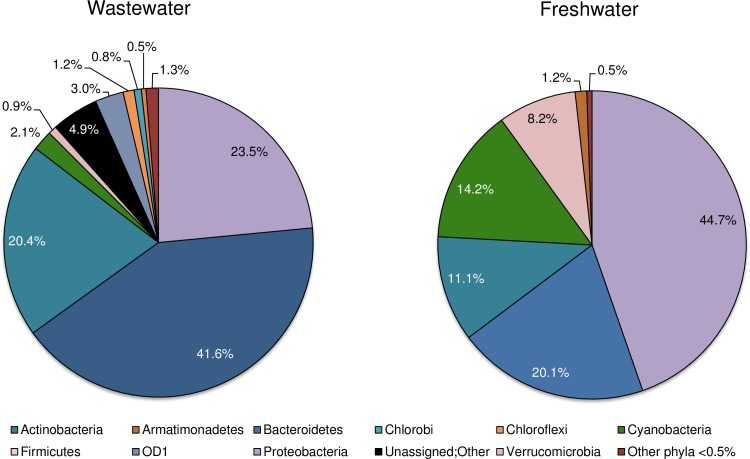
Composition of the main microbial phyla detected, by the universal primer set 515F_806R, in the wastewater and freshwater samples analysed during the study. For clarity, phyla forming < 0.5% abundance for each primer pair were combined.

For this study, a diverse range of environmental water sources, supposedly harbouring different microbial communities was chosen. To ensure that the assemblages were indeed different, an analysis of the results obtained by the universal primer set was performed. To this end, all the Bacterial and Archaeal phyla detected by the primers 515F_806R were pooled by water source (wastewater and freshwater) and compared (Fig 5; S4 File).

Overall, a greater range of the major bacterial phyla (n = 9) was observed in the wastewater samples, compared to freshwater (n = 5). Proteobacteria formed the largest proportion of bacterial phyla (34.1% on average, between the two water sources) but their average relative abundance clearly differed by water source, ranging from 44.7% in freshwater to 23.5% in wastewater. Bacteroidetes (30.8% on average, between the two water sources) and Actinobacteria (15.7%) were the second and third most abundant phyla, and were about twofold more abundant in wastewater than in freshwater. Interestingly the universal primer set detected Cyanobacteria at an average relative abundance of only 8.1%. This phylum was the fourth most abundant phylum overall, with values ranging from 14.2% in freshwater to only 2.1% in wastewater.

The fluctuations in the average relative abundance of the various taxa, detected by the universal primer set 515F_806R, confirm the dissimilarities in microbial composition, across the water samples chosen for the study.

### Comparison of primer performance with freshwater samples

**Fig 6:**
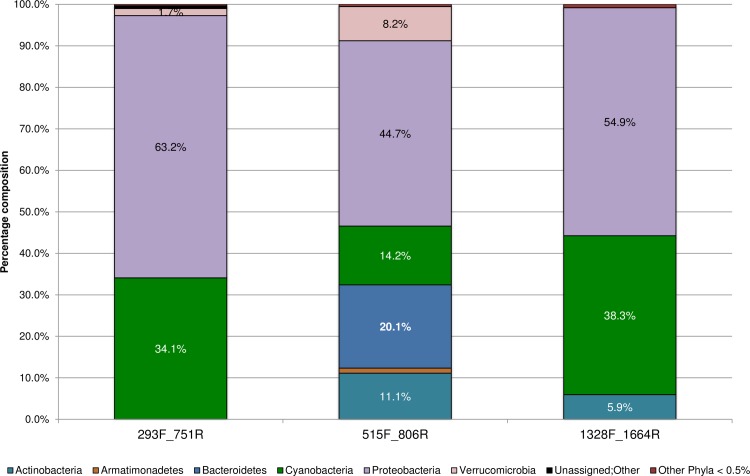
Composition of the main microbial phyla detected, by each primer set, in the freshwater samples analysed during the study. For clarity, phyla forming < 0.5% abundance for each primer pair were combined.

Due to the critical impact of bacterial contaminations on source water, a dedicated analysis was performed on the NGS data collected from freshwater samples only (Fig 6). Proteobacteria formed the largest proportion (≥ 44.7%) of the bacterial phyla sequenced by all primer pairs (Fig 6). The specificity of the novel primers is clear, with Cyanobacteria and Proteobacteria combined forming more than 95.3% of the total fraction of sequences generated by the 1328F_1664R and 293F_751R primers. This is in comparison to the universal 16S rDNA primers where reads from these two phyla combined formed only 58.9% of the total (Actinobacteria, Bacteroidetes and Verrucomicobia formed the major proportions of the remaining 41.1% of sequences). Apart from Verrucomicrobia (1.7% of the sequences generated by the 293F_751R primers) and Actinobacteria (5.9% of the sequences generated by the 1328F_1664R primers), all other phyla formed less than 1% of the total sequences generated, from freshwater, by the novel primers from the present study (Fig 6).

## Discussion

This study sought to design Cyanobacteria-specific 16S rDNA primers for use with NGS platforms, and to allow for rapid detection and identification of Cyanobacterial strains from a variety of environmental water samples. To this end, two different primer sets targeting different regions (V3, and V6 regions) of the 16S rDNA gene were designed and tested to compare their utility. This was performed in order to overcome the limitations posed by the use of universal 16S rDNA primers that were naturally designed to amplify across as broad a range as possible of bacterial and archaeal taxa.

The abundance of sequence data makes the 16S rDNA locus ideal for the design of phylum-specific primers and for the successive taxonomic assignment. *In silico–*and laboratory-tests performed during the present study confirmed that the novel primers have specificity for Cyanobacteria amplifying at least three times more Cyanobacterial sequences than the universal 16S rDNA primers (Fig 1, Table 3). Increased specificity for this phylum of algae is useful in water monitoring programs. For example, when there is the need to detect the presence of problematic Cyanobacteria present at low levels within complex multi-taxa aquatic communities whilst avoiding other non-problematic taxa. In these situations, the ability to enrich for members of a specific phylum of interest is clearly advantageous and we demonstrate this approach here together with the recovery of a broader range of Cyanobacterial orders when compared to the use of universal primers (Fig 4).

In addition to the preferential amplification of Cyanobacteria specific sequences, the specificity of the primers designed in this study was also directed toward the detection of Proteobacteria. Although the primers showed bias towards Proteobacteria sequences, they produced similar Proteobacterial profiles to that of the universal primers (Fig 4). This bias for Proteobacterial sequences was evident based upon the SILVA TestPrime results (Fig 1, esp. for 293F_751R), but was not observed in our initial primer specificity tests, or, when the primers were tested on Cyanobacterial cultures (i.e. sequencing results of the primers did not produce mixed signals, data not shown). However, amplification of Proteobacterial sequences by the 293F-751R and 1328F-1664R primer sets does not detract from their increased selectivity for Cyanobacterial sequences. This is best demonstrated in Fig 3 whereby these primers amplified more than twice as many Cyanobacterial sequences as compared to the universal 515F_806R primer set.

Proteobacteria are the largest and most phenotypically diverse bacterial division, accounting for the majority of known Gram-negative bacteria [53–55]. They form a complex phylum, arbitrarily divided into subdivisions based on their rRNA gene sequences, with pleomorphic members with various physiological and phenotypic characteristics, including numerous known human and animal pathogens [53, 55–57]. As such, the potential also exists to utilise these novel primers as a tool for detecting potentially pathogenic Proteobacteria from mixed communities.

For this study, the freshwater samples were obtained mainly from lakes/ ponds (lentic water systems). Future studies testing an even wider diversity of water sources (e.g. marine samples) or mock bacterial communities can be performed to better determine the applicability and limitations of these primers.

In specific applications where Cyanobacteria and Proteobacteria are considered the target groups, the increased specificity of the primers designed in this study allows for the pooling more samples in the same NGS run, while still guaranteeing adequate sequencing depth and spectrum of target taxa. This has obvious cost benefits. When combined with the faster sample processing and increased taxonomic resolution, this makes NGS cheaper, and faster, than current traditional morphology-based taxonomic methods of bioassessment [14]. The ever reducing cost per base of sequencing [3, 58], together with the reproducibility and potential for standardisation [8] make NGS a potential tool for biomonitoring [12, 19, 26, 59]. When Cyanobacteria and Proteobacteria are important target taxa to monitor, the increased selectivity of the novel primers should allow parallel sequencing of at least twice the number of samples, per each run, compared to the universal primers. This should allow a reduction of the NGS costs, yet guaranteeing optimal coverage and species discrimination.

## Supporting Information

S1 Fig Cyanobacteria targeted primers as mapped onto *Escherichia coli*. Screen capture from Geneious (Biomatters, NZ) showing cyanobacteria 16S rDNA consensus sequence mapped onto E. coli K-12 substrain MG1655 (NR10284) Annotations indicate the positions of the hypervariable regions (blue boxes), and primer binding sites. Black arrows indicate positions of reference primers, green/grey arrows indicate the positions of primers designed in this study.
(DOCX)

S2 Fig Rarefaction plots showing OTUs observed by primer set. Rarefaction of sampled OTUs observed by the primer sets used in the study. A sampling depth of 205 taxonomic counts per sample was used to ensure inclusion of all samples used in the study.
(DOCX)

S1 File Detailed relative abundance of phyla detected by each primer set used in the study. (XLSX)

S2 File Detailed relative abundance of cyanobacterial sequences detected by each primer set used in the study. Class and order, together with the total number of genera detected by all primer sets are indicated in the left-most column.
(XLSX)

S3 File Detailed relative abundance of proteobacterial sequences detected by each primer set used in the study. Class and order, together with the total number of genera detected by all primer sets are indicated in the left-most column.
(XLSX)

S4 File Relative abundance of phyla in the environmental water samples as amplified by each primer set used in the study. Breakdown of the different phyla detected in each water source by each primer set used in the study.
(XLSX)
