# Supplementary material for: Novel Primer Sets for Next Generation Sequencing-Based Analyses of Water Quality
Source: PLoS One. 2017 Jan 24;12(1):e0170008. doi: 10.1371/journal.pone.0170008 (PMC5261608; doi:10.1371/journal.pone.0170008)
Supplement: S1 Fig — Screen capture from Geneious (Biomatters, NZ) showing cyanobacteria 16S rDNA consensus sequence mapped onto E. coli K-12 substrain MG1655 (NR10284) Annotations indicate the positions of the hypervariable regions (blue boxes), and primer binding sites. Black arrows indicate positions of reference primers, green/grey arrows indicate the positions of primers designed in this study. (DOCX) [file pone.0170008.s001.docx]

S1 Fig. Cyanobacteria targeted primers as mapped onto *Escherichia coli*


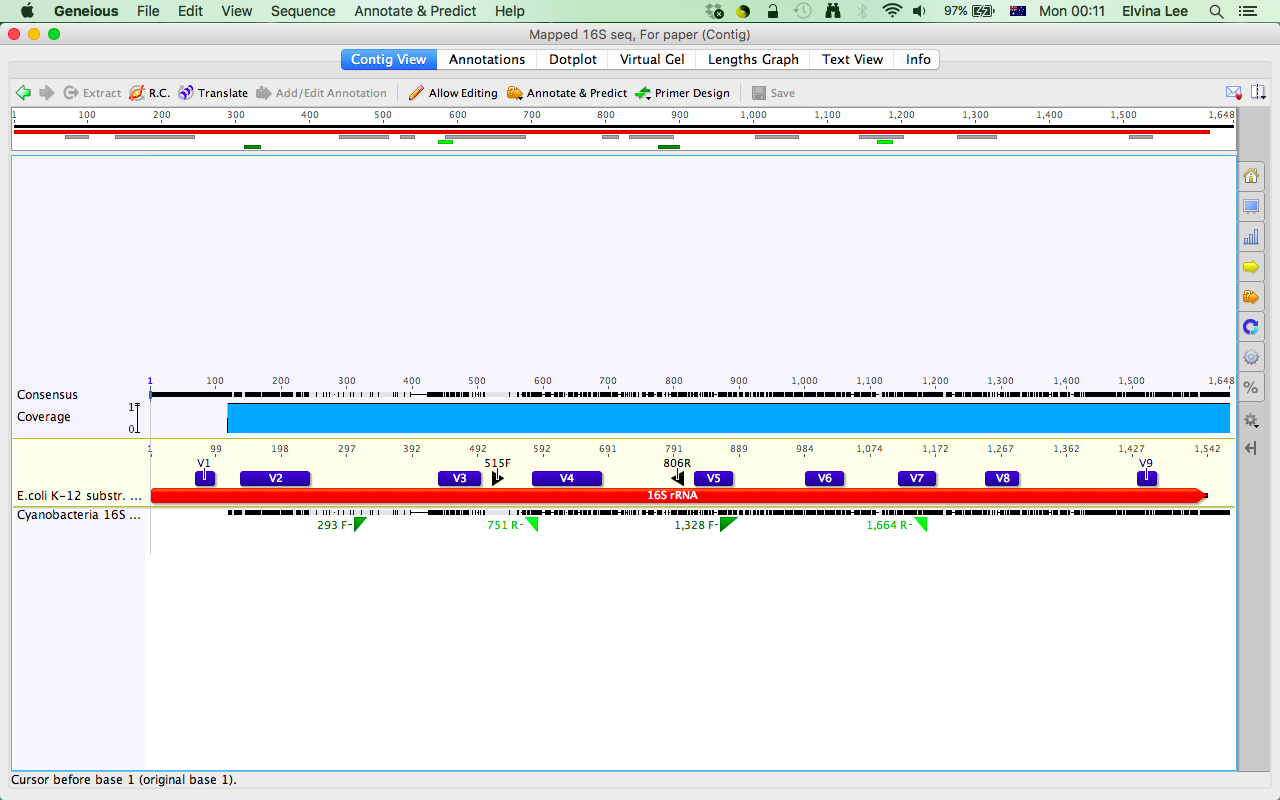


Figure 1: Screen capture from Geneious (Biomatters, NZ) showing cyanobacteria 16S rDNA consensus sequence mapped onto *E. coli* K-12 substrain MG1655 (NR10284) Annotations indicate the positions of the hypervariable regions (blue boxes), and primer binding sites. Black arrows indicate positions of reference primers, green/grey arrows indicate the positions of primers designed in this study.
