# Supplementary material for: Novel Primer Sets for Next Generation Sequencing-Based Analyses of Water Quality
Source: PLoS One. 2017 Jan 24;12(1):e0170008. doi: 10.1371/journal.pone.0170008 (PMC5261608; doi:10.1371/journal.pone.0170008)
Supplement: S2 Fig — Rarefaction of sampled OTUs observed by the primer sets used in the study. A sampling depth of 205 taxonomic counts per sample was used to ensure inclusion of all samples used in the study. (DOCX) [file pone.0170008.s002.docx]

S2 Fig. Rarefaction plots showing OTUs observed by primer set.


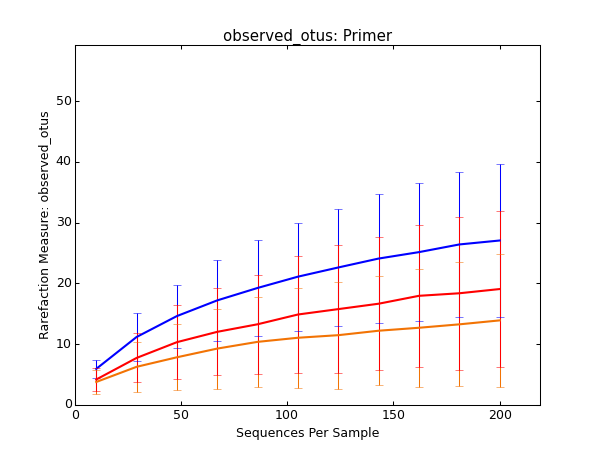


Figure 1: Rarefaction of sampled OTUs observed by the primer sets used in the study. A sampling depth of 205 taxonomic counts per sample was used to ensure inclusion of all samples used in the study.
